# Middle East Respiratory Syndrome Coronavirus NS4b Protein Inhibits Host RNase L Activation

**DOI:** 10.1128/mBio.00258-16

**Published:** 2016-03-29

**Authors:** Joshua M. Thornbrough, Babal K. Jha, Boyd Yount, Stephen A. Goldstein, Yize Li, Ruth Elliott, Amy C. Sims, Ralph S. Baric, Robert H. Silverman, Susan R. Weiss

**Affiliations:** a Department of Microbiology, Perelman School of Medicine at the University of Pennsylvania, Philadelphia, Pennsylvania, USA; b Department of Cancer Biology, Lerner Research Institute, Cleveland Clinic, Cleveland, Ohio, USA; c Department of Epidemiology, University of North Carolina at Chapel Hill, Chapel Hill, North Carolina, USA; d Department of Microbiology and Immunology, University of North Carolina at Chapel Hill, Chapel Hill, North Carolina, USA

## Abstract

Middle East respiratory syndrome coronavirus (MERS-CoV) is the first highly pathogenic human coronavirus to emerge since severe acute respiratory syndrome coronavirus (SARS-CoV) in 2002. Like many coronaviruses, MERS-CoV carries genes that encode multiple accessory proteins that are not required for replication of the genome but are likely involved in pathogenesis. Evasion of host innate immunity through interferon (IFN) antagonism is a critical component of viral pathogenesis. The IFN-inducible oligoadenylate synthetase (OAS)-RNase L pathway activates upon sensing of viral double-stranded RNA (dsRNA). Activated RNase L cleaves viral and host single-stranded RNA (ssRNA), which leads to translational arrest and subsequent cell death, preventing viral replication and spread. Here we report that MERS-CoV, a lineage C *Betacoronavirus*, and related bat CoV NS4b accessory proteins have phosphodiesterase (PDE) activity and antagonize OAS-RNase L by enzymatically degrading 2′,5′-oligoadenylate (2-5A), activators of RNase L. This is a novel function for NS4b, which has previously been reported to antagonize IFN signaling. NS4b proteins are distinct from lineage A *Betacoronavirus* PDEs and rotavirus gene-encoded PDEs, in having an amino-terminal nuclear localization signal (NLS) and are localized mostly to the nucleus. However, the expression level of cytoplasmic MERS-CoV NS4b protein is sufficient to prevent activation of RNase L. Finally, this is the first report of an RNase L antagonist expressed by a human or bat coronavirus and provides a specific mechanism by which this occurs. Our findings provide a potential mechanism for evasion of innate immunity by MERS-CoV while also identifying a potential target for therapeutic intervention.

## IMPORTANCE

Middle East respiratory syndrome coronavirus (MERS-CoV) is the first highly pathogenic human coronavirus to emerge since severe acute respiratory syndrome coronavirus (SARS-CoV). MERS-CoV, like other coronaviruses, carries genes that encode accessory proteins that antagonize the host antiviral response, often the type I interferon response, and contribute to virulence. We found that MERS-CoV NS4b and homologs from related lineage C bat betacoronaviruses BtCoV-SC2013 (SC2013) and BtCoV-HKU5 (HKU5) are members of the 2H-phosphoesterase (2H-PE) enzyme family with phosphodiesterase (PDE) activity. Like murine coronavirus NS2, a previously characterized PDE, MERS NS4b, can antagonize activation of the OAS-RNase L pathway, an interferon-induced potent antiviral activity. Furthermore, MERS-CoV mutants with deletion of genes encoding accessory proteins NS3 to NS5 or NS4b alone or inactivation of the PDE can activate RNase L during infection of Calu-3 cells. Our report may offer a potential target for therapeutic intervention if NS4b proves to be critical to pathogenesis in *in vivo* models of MERS-CoV infection.

## INTRODUCTION

Middle East respiratory syndrome coronavirus (MERS-CoV) infections range from mild upper respiratory infections to severe acute respiratory distress syndrome, with a global case fatality rate of 36% (1, 2). MERS-CoV has predominantly affected the Kingdom of Saudi Arabia and neighboring countries with sporadic cases arising in Europe and North America as the result of travel to and from the Middle East (3). A recent outbreak of MERS-CoV in South Korea has raised the specter that unrecognized infections combined with potential superspreaders may pose a much greater risk of significant travel-associated outbreaks of MERS-CoV than previously suspected, particularly in health care settings (4, 5). The lethality of MERS-CoV and the ease of global travel necessitate further study and understanding of the mechanisms of MERS-CoV pathogenesis.

MERS-CoV, a lineage C *Betacoronavirus*, has a 30-kb positive-sense single-stranded RNA (ssRNA) genome. As with all CoVs, the first 5′ two-thirds of the genome consists of the replicase encoded in open reading frame 1a (ORF1a) and ORF1b. The remaining 3′ one-third encodes the structural proteins spike (S), envelope (E), membrane (M), and nucleocapsid (N) as well as accessory proteins (encoded in ORF3 to ORF5 and ORF8b) that are not required for genome replication but likely act as immune antagonists that may be critical for pathogenesis (6–8). Indeed, MERS-CoV and other CoVs induce type I interferon (IFN) only weakly and late in infection (9–15), suggesting that coronaviruses have evolved mechanisms of immune evasion. Furthermore, there are reports that MERS-CoV accessory protein NS4a (encoded by ORF4a), NS4b (encoded by ORF4b), and NS5 (encoded by ORF5), when overexpressed from plasmids, antagonize type I IFN induction and signaling at various points in the pathways (16–19). NS4a is a double-stranded RNA (dsRNA) binding protein (17). NS4b is detected primarily in the nucleus (16–19), making NS4b and the severe acute respiratory syndrome coronavirus (SARS-CoV) ORF3a-encoded proteins (20, 21) the only known coronavirus proteins thus far to be detected in the nucleus (20, 21). A MERS-CoV mutant with deletions of ORFs 3 to 5 is attenuated for replication in human airway-derived Calu-3 cells (22), and a mutant with deletions of ORFs 4a and 4b is attenuated for replication in hepatic carcinoma-derived Huh-7 cells (23).

The type I IFN response is an essential component of antiviral innate immunity and is initiated when host sensors initiate signaling pathways resulting in transcription of type I IFN genes (9, 10, 24). IFN activates transcription of numerous IFN-stimulated genes (ISG); among these and most relevant to this report are the *OAS* genes (25). Oligoadenylate synthetase (OAS), upon detection and binding of dsRNA, synthesizes 2′,5′-oligoadenylate (2-5A) [p*_x_*5′A(2′p5′A)_n_; *x* = 1 to 3; *n* ≥ 2] from intracellular ATP that induces the homodimerization of latent RNase L, leading to its subsequent activation (24, 26, 27). Activated RNase L cleaves both viral and host ssRNA preferentially at UU and UA dinucleotide sequences, leading to translational arrest and apoptosis, and limits viral replication and spread *in vitro* and *in vivo* (24, 28, 29). In addition, RNA cleavage products can be recognized by RNA sensors, leading to further augmentation of IFN production and signaling (30).

We have shown previously that lineage A *Betacoronavirus* mouse hepatitis virus (MHV) NS2 is a determinant of cellular and organ tropism. MHV NS2 is a 2′,5′-phosphodiesterase (PDE) that antagonizes the type I IFN response by blocking activation of the OAS-RNase L pathway and is a critical determinant of MHV hepatovirulence (7, 29). Here we report that by structural homology, biochemistry, and biological measures, MERS-CoV NS4b and homologs encoded by related bat lineage C *Betacoronavirus*, BtCoV-SC2013 (SC2013) and BtCoV-HKU5 (HKU5), are also 2′,5′-PDEs that can antagonize the IFN-inducible OAS-RNase L pathway.

## RESULTS

### Alignment and modeling of NS4b proteins.

**FIG 1:**
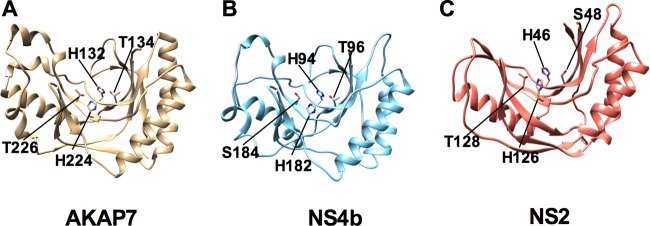
Predicted structure of MERS-CoV NS4b phosphodiesterase. (A) Structure of *Rattus norvegicus* AKAP7γ/δ (PDB: 2VFK) (37). (B) Tertiary structural homology model of MERS-CoV NS4b. (C) Structure of NS2 (PDB: 4Z5V) (36). The structures were visualized and analyzed in UCSF Chimera 1.8.

To elucidate the function of MERS-CoV NS4b, we queried the primary amino acid sequence with the National Center for Bioinformatics (NCBI) Basic Local Alignment Search Tool (BLAST). MERS-CoV NS4b was found to have no primary amino acid sequence homology outside of lineage C *Betacoronavirus*. To broaden the search for homologous proteins, we used tertiary structural prediction and homology search by Phyre^2^ (31) and I-TASSER (32, 33). The top scoring structure identified by both servers was the central domain of *Rattus norvegicus* A kinase anchoring protein 7 isoform gamma or delta (AKAP7γ/δ) (PDB: 2VFK), a 2H-phosphoesterase (2H-PE) superfamily member with 2′,5′-PDE activity (34) (Fig. 1A). These enzymes are characterized by two H-Φ-[ST]-Φ motifs (where Φ is a hydrophobic residue) separated by an average of 80 residues (35). To generate a more accurate predicted structure, the 2H-PE domain of MERS-CoV NS4b was modeled directly on AKAP7 using one-to-one threading on Phyre^2^ followed by loop and side chain refinement in Modeller (Fig. 1B) (29, 31). For comparison, the recently solved structure of lineage A *Betacoronavirus* mouse hepatitis virus (MHV) NS2, a 2H-PE with 2′,5′-PDE activity, is also shown (Fig. 1C) (36).

**FIG 2:**
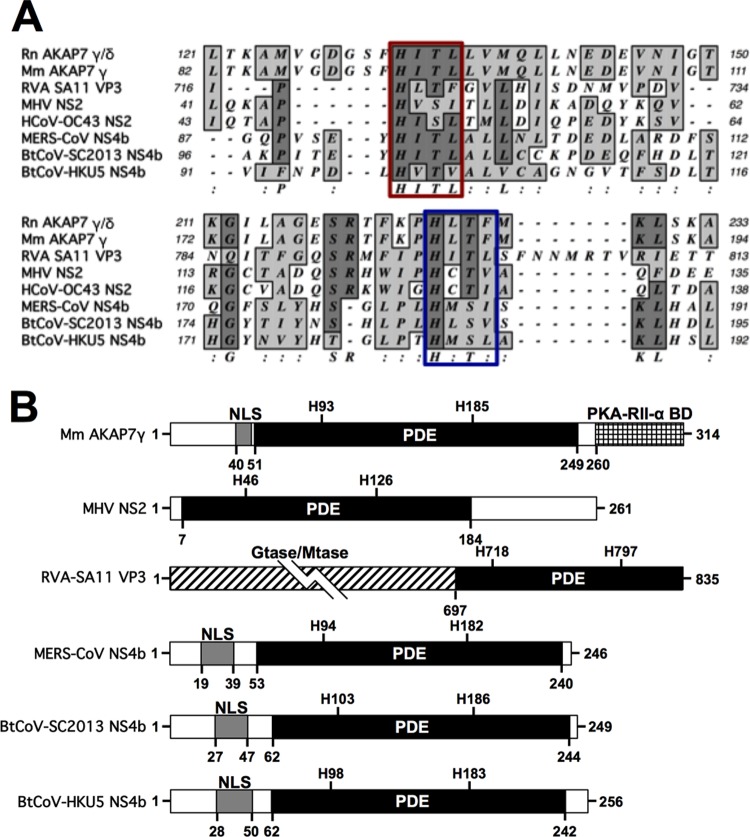
Cellular and viral 2′,5′-phosphodiesterase (PDE) domains. (A) Alignment of known and predicted cellular and viral 2′,5′-PDE sequences. Catalytic motifs [H-Φ-(S/T)-Φ] are indicated by the red and blue boxes. Rat (*Rattus norvegicus* [Rn]) and mouse (*Mus musculus* [Mm]) AKAP sequences and human (H) and bat (Bt) coronavirus sequences are shown. (B) Comparison of known features of full-length *M. musculus* AKAP7γ, MHV NS2, RVA VP3 CTD and lineage C NS4b proteins including nuclear localization sequence (NLS) and PDE domains. PKA-RII-α-BD, a binding domain for regulatory subunit (RII) of cAMP-dependent protein kinase A (37**)**, guanylyltransferase (Gtase), and methyltransferase (Mtase) domains are also indicated (32).

MERS-CoV NS4b shares significant homology with closely related bat coronavirus (BtCoV) proteins encoded by SC2013 and HKU5, 47% and 31% amino acid sequence homology, respectively. While both BtCoV homologs have the 2 [H-Φ-(S/T)-Φ] predicted catalytic motifs, SC2013 NS4b (previously referred to as NS3c [37]) had low-confidence structural homology (<45%) with AKAP7, and HKU5 NS4b had no significant structural homology with any structures in the Phyre^2^ database (38). However, alignment of the regions surrounding the H-Φ-(S/T)-Φ motifs with cellular AKAP7 from rat, mouse (*Mus musculus*, NP_061217.3), and virus-encoded 2′,5′-PDEs illustrates the conservation of these two motifs (Fig. 2A). We have previously shown that the central domain of the *M. musculus* AKAP7γ and simian rotavirus A (RVA), strain SA11, VP3 protein carboxy-terminal domain (CTD) are catalytically active 2′,5′-PDEs (28, 34, 35, 39). Additionally, MERS-CoV NS4b and BtCoV homologs, like the rat and mouse AKAP7 but unlike any of the known viral PDEs, including MHV NS2 or rotavirus VP3 CTD, contain an amino-terminal nuclear localization signal (NLS) (Fig. 2B) (16, 18, 19). In addition to a PDE domain, AKAP7γ also contains a carboxy-terminal binding domain for the regulatory subunit II (RII) of cyclic AMP (cAMP)-dependent protein kinase A (PKA) (PKA-RII-α-BD) (39), and VP3 contains amino-terminal guanylyltransferase (Gtase) and methyltransferase (Mtase) domains (E) (28), neither of which are found adjacent to coronavirus PDE domains.

### NS4b from MERS-CoV and both BtCoVs are enzymatically active 2′,5′-phosphodiesterases that cleave 2-5A.

**FIG 3:**
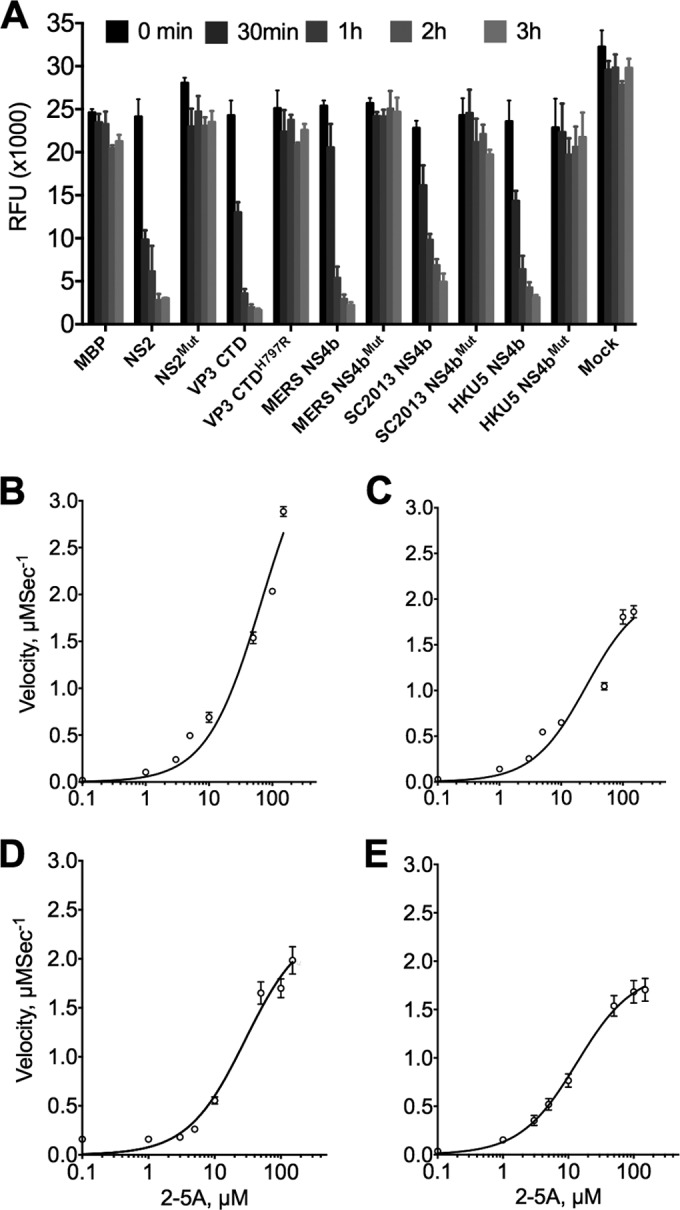
Lineage C NS4b proteins are catalytically active 2′,5′-phosphodiesterases. (A) Abrogation of catalytic activity by mutation of the second catalytic His of NS4b proteins and comparison with MHV NS2 and RVA VP3 CTD. The kinetic data were obtained using 10 µM 2-5A and 1 µM enzyme concentration with the exception of MBP, which was used at 10 µM. Data shown are from one representative experiment of three carried out with separate enzyme preparations. (B to D) Steady-state enzyme kinetic curves of velocity versus concentration of 2-5A for MHV NS2 (B), MERS-CoV (C), BtCov-SC2013 (D), and BtCoV-HKU5 (E) NS4b proteins. Kinetic reactions were carried out in triplicate and expressed as means ± standard errors of the means (SEM) (error bars). Substrate-dependent velocity measurements are shown from one representative experiment carried out twice in triplicate with separate enzyme preparations.

To determine whether MERS-CoV, BtCoV-SC2013, and BtCoV-HKU5 NS4b encode enzymatically active PDEs, NS4b genes were expressed in *Escherichia coli* as maltose binding protein (MBP) fusion proteins and purified by affinity chromatography followed by ion exchange and size exclusion chromatography. Purified proteins were incubated with various 2-5A substrate concentrations and subsequently used to assess enzyme kinetics via a fluorescence resonance energy transfer (FRET)-based RNase L activation assay (29). Substrate steady-state kinetic studies were performed (see Materials and Methods for details), and the quantity of cleaved 2-5A was determined as a function of time from 180-min progress curves conducted in triplicate. The best-fit parameter estimates are shown in Table 1. All three proteins were capable of cleaving 2-5A and preventing the activation of RNase L with similar kinetic parameters to MHV NS2. As expected, mutant proteins containing a His-to-Arg mutation in the second catalytic motif were incapable of cleaving 2-5A (Fig. 3A). Although the activities of all three NS4b proteins were similar to that of MHV NS2 (Fig. 3B), velocities plateaued at lower concentrations of 2-5A compared with MHV NS2, suggesting that the lineage C proteins become saturated more quickly at higher 2-5A concentrations (Fig. 3C to E).

**TABLE 1:** Enzyme kinetic parameters^a^

Enzyme	ppp5′A2′p5′A2′p5′A (2-5A substrate)		
	*k*_cat_ (s^−1^)	*Km* (µM)	*k*_cat_/*Km* (μM^−1^ s^−1^)
MHV NS2	3.9 ± 0.4	6.7 ± 1.6	0.6
MERS-CoV NS4b	2.1 ± 0.2	2.6 ± 0.6	0.8
SC2013 NS4b	2.4 ± 0.1	2.9 ± 0.6	0.8
HKU5 NS4b	1.9 ± 0.1	1.3 ± 0.3	1.4

a The enzyme kinetic parameters were determined by GraphPad Prism by the equation *Y* = Et × *k*_cat_ × [X/(*Km* + *X*)], where *Y* is the velocity of reaction in micromolar per second, *X* is the substrate concentration in micromolar, and Et is the concentration of enzyme catalytic sites. The kinetics were determined in triplicate reactions, and data are expressed as means ± standard errors of the means.

### Expression of MERS-CoV NS4b and BtCoV homologs from chimeric MHV.

**FIG 4:**
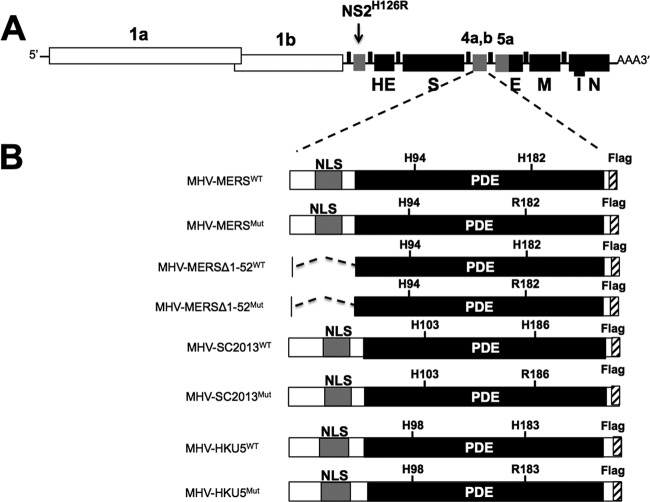
Construction of NS4b-expressing recombinant MHV. (A) Diagrammatic representation of MHV^Mut^ genome (expressing catalytically inactive NS2^H126R^). The letters designate genes encoding structural proteins, and the numbers designate ORFs encoding nonstructural proteins. ORFs 1a and 1b together comprise 20 kb and are not to scale. (B) Wild-type or catalytic mutant NS4b genes are lined up with the MHV ORF4a or ORF4b insertion site to construct chimeric viruses. Nuclear localization signals (NLSs) and phosphodiesterase (PDE) domains are indicated.

**FIG 5:**
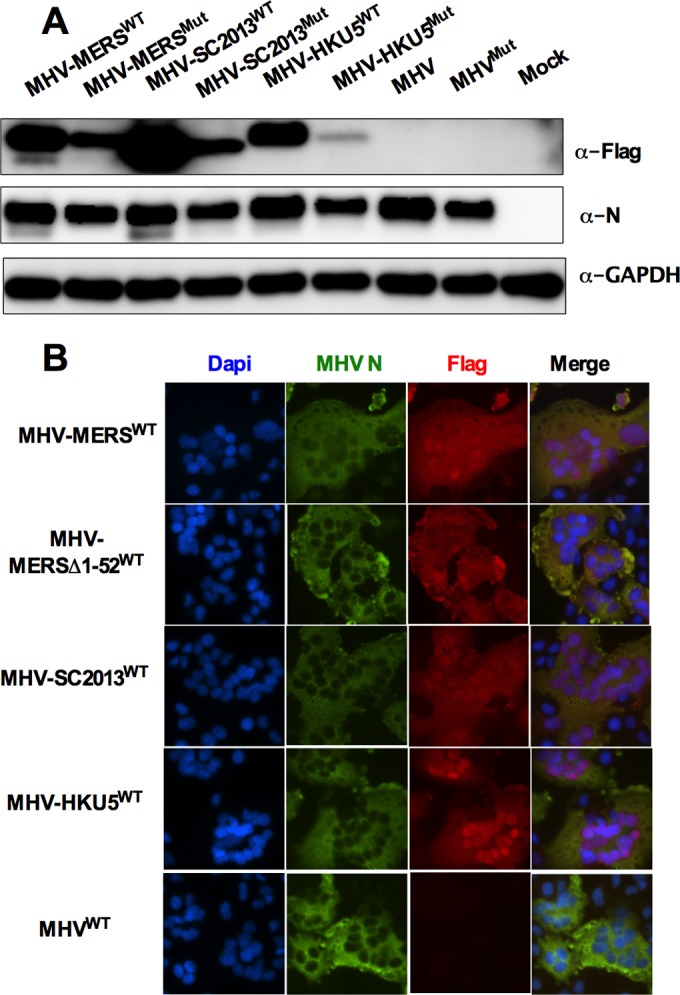
Expression and localization of MERS-CoV NS4b and BtCoV homologs expressed by chimeric MHVs. (A) Lysates of chimeric-virus-infected RNase L^−/−^ BMM were analyzed on denaturing polyacrylamide gels and analyzed by immunoblotting with antibody against Flag (α-Flag) to detect NS4b, antibody against MHV nucleocapsid (N) protein, and antibody against GAPDH. Data are representative of three experiments carried out twice in 17Cl-1 cells and once each in B6 and RNase L^−/−^ BMM with similar results. (B) Murine L2 cells infected with chimeric MHVs were fixed and stained with DAPI as well as antibodies against Flag to detect NS4b and MHV N. These data are from one representative experiment of three.

To investigate OAS-RNase L antagonism in cell culture, using the backbone of catalytically inactive NS2 with the H126R substitution (NS2^H126R^) (MHV^Mut^), we constructed chimeric MHVs that express one of the NS4b proteins, and for each chimeric MHV, we constructed a corresponding catalytically inactive mutant, each with a carboxy-terminal Flag tag (18, 34). In addition, we constructed chimeric MHVs expressing MERS-NS4b with a deletion of the amino-terminal 52 amino acids including the NLS (MHV-MERSΔ1-52^WT^) as well as its catalytically inactive mutant MHV-MERSΔ1-52^Mut^. Thus, wild-type (WT) or mutant NS4b genes were cloned into the MHV^Mut^ genome in place of MHV ORFs 4a and 4b which encode protein(s) with no known function in MHV replication or pathogenesis (40) (Fig. 4). To assess expression of NS4b proteins from chimeric viruses, bone marrow-derived macrophages (BMM) derived from mice genetically deficient in RNase L (RNase L^−/−^) were infected with each of the chimeric viruses in addition to WT MHV and MHV^Mut^ expressing catalytically inactive mutant NS2. (RNase L^−/−^ BMM were used rather than B6 BMM because an active PDE is required for robust replication in the latter [29].) BMM were lysed at 12 h postinfection, and protein lysates were analyzed by Western immunoblotting with antibodies directed against Flag to detect NS4b proteins, antibodies against viral nucleocapsid to assess viral replication, and antibodies against glyceraldehyde-3-phosphate dehydrogenase (GAPDH) as a loading and transfer control. All NS4b proteins were expressed in RNase L^−/−^ BMM but to different extents, and mutant NS4b proteins were expressed at lower levels than WT NS4b proteins were, possibly reflecting reduced protein stability as a result of the mutations (Fig. 5A). Similar findings were obtained with chimeric virus infection of 17Cl-1 cells (data not shown).

All three NS4b proteins have an amino-terminal tripartite NLS; previous studies reported that MERS-CoV NS4b is predominantly detected in the nucleus (41) and that overexpressed BtCoV-HKU5 NS4b is solely detected in the nucleus (16). Previously, we found that murine AKAP7 was exclusively nuclear and that it could not act as a PDE to rescue replication of chimeric mutant MHV unless the NLS was removed and cytoplasmic expression was achieved (34), so it was important to determine the subcellular localization of NS4b protein during chimeric virus infection. Thus, immunofluorescence staining was carried out to assess expression and subcellular localization of expression of each NS4b protein. Murine L2 cells were infected with chimeric viruses expressing both MERS-CoV NS4b and corresponding CoV homologs, fixed at 8 h postinfection, and stained with anti-Flag antibodies (NS4b) and antibodies against the MHV nucleocapsid protein, which is expressed in the cytoplasm (Fig. 5B). Interestingly, MERS-CoV and BtCoV-SC2013 NS4b proteins localized to the nucleus with expression in the cytoplasm as well, while the BtCoV-HKU5 NS4b homolog was almost completely nuclear, similar to reports of overexpressed NS4b in the literature (16–18). As expected, MHV-MERSΔ1-52^WT^ expressing an NS4b with deletion of the NLS was localized to the cytoplasm.

### NS4b proteins rescue replication of MHV^Mut^ in B6 BMM by antagonizing RNase L.

**FIG 6:**
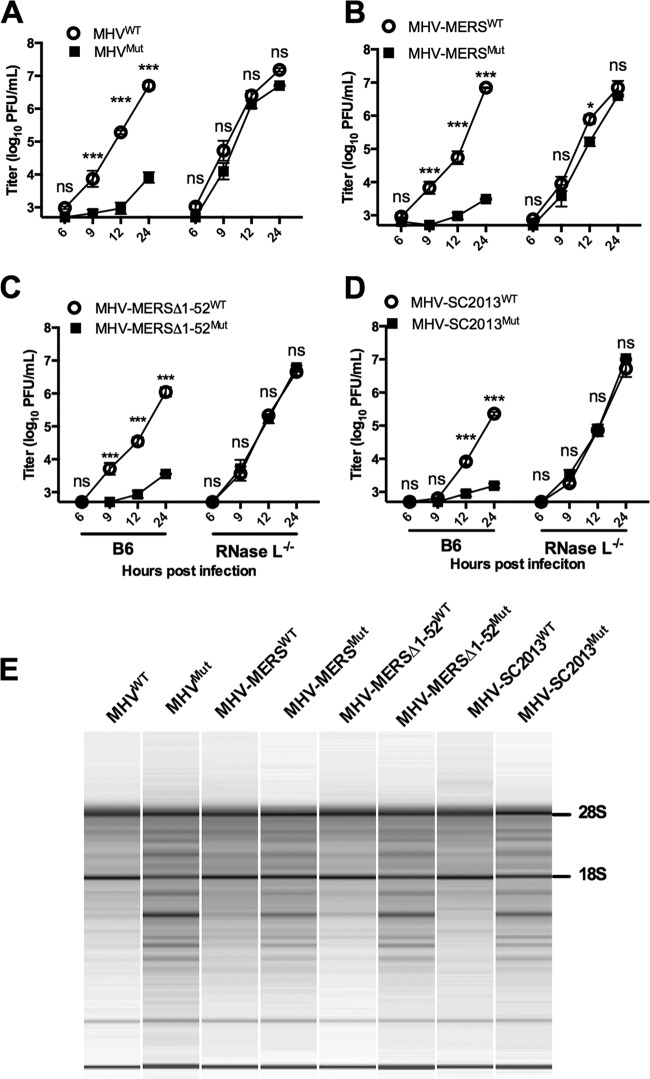
Lineage C PDE NS4b proteins functionally replace MHV NS2 in primary BMM. (A to D) Representative one-step growth curve of WT and mutant (Mut) MHV (A) and chimeric viruses expressing WT and mutant NS4b proteins (diagrammed in Fig. 4B), WT and mutant MHV-MERS (B), WT and mutant MHV-MERSΔ1-52 (C), and WT and mutant MHV-SC2013 (D) in B6 and RNase L^−/−^ BMM (MOI of 1 PFU; *n* = 3). Statistical significance was determined by two-way analysis of variance (ANOVA) with Sidak’s multiple comparisons and is indicated as follows: *, *P* value of <0.05; **, *P* < 0.01; ***, *P* < 0.001. Values that are not significantly different (ns) are indicated. Values are means ± SEM (error bars). These data are from one representative experiment of three experiments. (E) rRNA degradation pattern 15 h postinfection in B6 BMM. Data are from one representative experiment of three (MOI of 1 PFU; *n* = 3). The positions of 28S and 18S rRNAs are shown to the right of the gel.

NS2 expression is necessary for robust replication of MHV in BMM derived from B6 BMM (WT) but is not required in RNase L^−/−^ BMM (7, 29). To elucidate the roles of the NS4b proteins in the context of viral infection, we inoculated B6 BMM or RNase L^−/−^ BMM with wild-type MHV (MHV^WT^) or MHV expressing a catalytically inactive NS2 (MHV^Mut^) as well as chimeric MHV^Mut^ expressing either WT or mutant MERS-CoV (both full-length and with amino acids 1 to 52 deleted) and BtCoV-SC2013 NS4b proteins (see Fig. 4 for schematics of the chimeric viruses). All three WT NS4b proteins, but not mutant NS4b proteins, were able to rescue MHV^Mut^ replication in WT BMM (Fig. 6A to D). Similar results were obtained for the pair of viruses expressing BtCoV HKU5 NS4b proteins (data not shown). MHV expressing MERS-CoV WT NS4b (MHV-MERS^WT^) replicated to a titer equivalent to that of WT MHV. Viruses expressing the BtCoV homologs replicated well in B6 BMM but not quite to the same extent as WT MHV or MHV-MERS^WT^, similar to findings with the chimeric viruses expressing PDE of RVA VP3 CTD and cellular AKAP7 protein (28, 34) (Fig. 6D and data not shown). As expected, viruses expressing mutant as well as WT NS4b proteins replicated to high titer in RNase L^−/−^ BMM. Although localization of NS4b proteins was primarily nuclear, these results demonstrate that the level of expression of NS4b PDE at low levels in the cytoplasm was sufficient to rescue replication in MHV-MERS- and MHV-SC2013-infected B6 BMM.

rRNA degradation during infection is indicative of RNase L activation, and we have previously used this read out as an indirect measure of RNase L activation (29). In addition, we previously demonstrated that enzymatically active PDEs of RVA VP3 and AKAP7, but not catalytically inactive mutant proteins, were capable of preventing rRNA degradation in B6 BMM during MHV chimeric virus infection as determined by analyzing total RNA levels postinfection on an Agilent bioanalyzer. Similarly, MHV-MERS^WT^, both full-length and MHV-MERSΔ1-52^WT^ mutant and MHV-SC2013^WT^ clearly prevented RNase L-mediated RNA degradation, while viruses expressing the inactive mutant proteins activated the OAS-RNase L pathway. Surprisingly, BtCOV-HKU5^WT^ (expressed during MHV-HKU5^WT^ infection) was unable to efficiently prevent rRNA degradation despite the fact that it did confer efficient replication in B6 BMM (data not shown). This may be due to the mostly nuclear localization of MHV-HKU5^WT^, which is discussed further below.

### MERS-CoV NS4b rescues replication of MHV^Mut^ *in vivo* in B6 mice.

**FIG 7:**
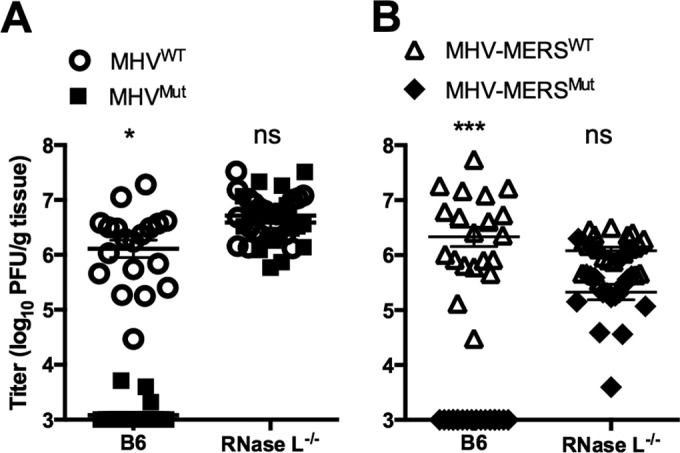
Catalytically active MERS-CoV NS4b rescues replication of MHV^Mut^ in the livers of B6 mice. Liver titers (*n* = 19 or 20) day 5 postinfection (MOI of 2,000 PFU) of MHV^WT^ and MHV^Mut^ (A) or MHV-MERS^WT^ and MHV-MERS^Mut^ (B). Statistical significance was determined by χ^2^ test with Yates’ correction and is indicated as follows: *, *P*  value of <0.05; ***, *P* < 0.001. Values are means ± SEM (error bars). Values that are not significantly different (ns) are indicated. These data are pooled from four experiments

To determine whether MERS-CoV NS4b was capable of rescuing replication of MHV^Mut^ *in vivo*, we infected B6 and RNase L^−/−^ mice with chimeric viruses expressing either MHV-MERS^WT^ or enzymatically inactive virus (MHV-MERS^Mut^) and compared replication to MHV^WT^. NS4b, but not NS4b^Mut^, rescued MHV^Mut^ replication to MHV^WT^ levels in B6 mice (Fig. 7). Additionally, gross pathology was restored for MHV-MERS^WT^, which is typically fully abrogated without an enzymatically active NS2, but not for MHV-MERS^Mut^ (data not shown). This confirms that MERS-CoV NS4b can act as an RNase L antagonist and contribute to pathogenesis *in vivo*.

### MERS-CoV inhibits rRNA degradation in a human airway cell line.

**FIG 8:**
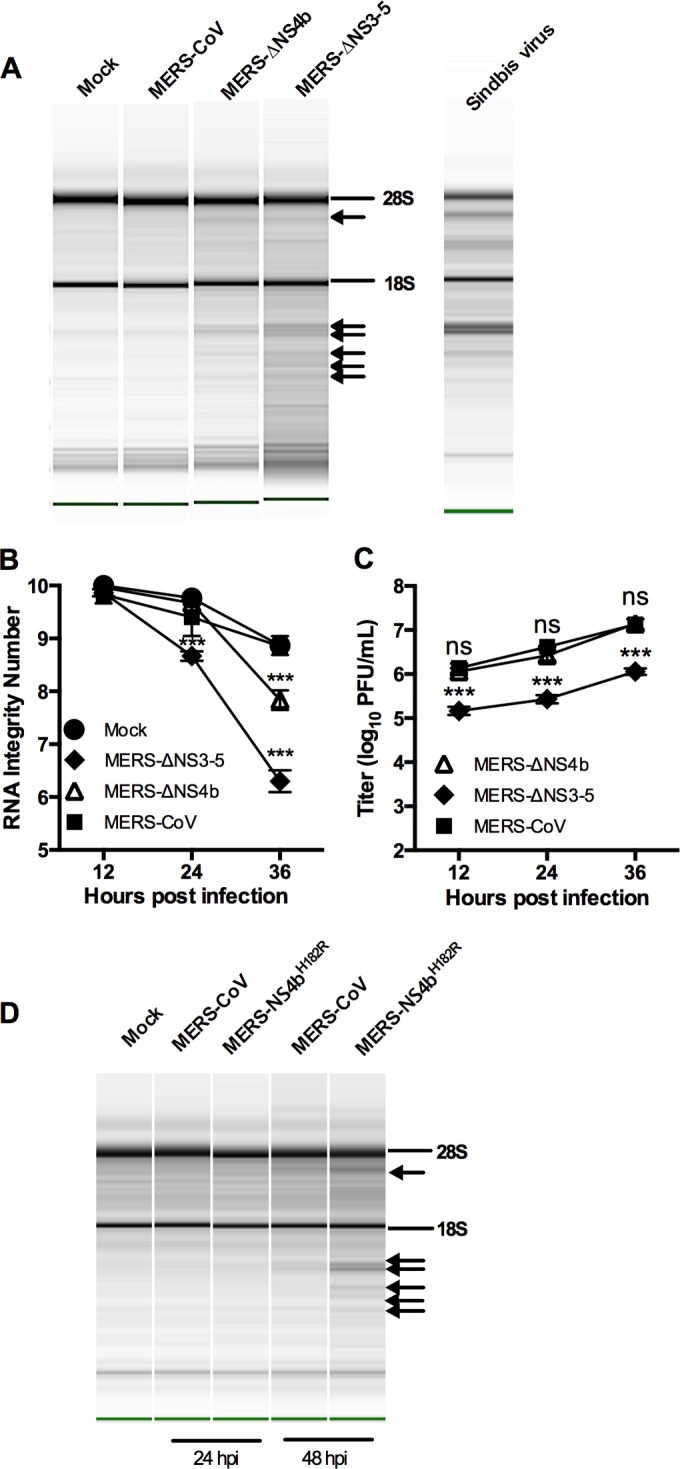
MERS-CoV inhibits rRNA degradation in human airway cells. (A to C) rRNA degradation pattern from cells 36 h postinfection (A), quantification by RNA integrity number (RIN) (B), and replication kinetics (C) of mock-infected and MERS-CoV-, MERS-ΔNS4b-, and MERS-ΔNS3-5-infected Calu-3 cells (MOI of 1 PFU; *n* = 3). Statistical significance was determined by two-way ANOVA with Sidak’s multiple comparisons and indicated as follows: ***, *P* value of <0.001; ns, not significant. Values are means ± SEM (error bars). RNA from Sindbis virus-infected human A549 cells (run separately on the Agilent Bioanalyzer) is shown in panel A as a marker for the RNase L-induced pattern of degradation of human rRNA. (D) rRNA degradation pattern from cells 24 and 48 h after MERS-NS4b^H182R^ infection of Calu-3 cells. The positions of 28S and 18S rRNAs are indicated in panels A and D. Data shown are from one representative experiment of two.

We constructed a MERS-CoV NS4b deletion mutant (MERS-ΔNS4b) and mock inoculated or inoculated a human airway epithelial cell line, Calu-3, with MERS-CoV, MERS-ΔNS4b, or MERS-CoV lacking NS3 to NS5 (MERS-ΔNS3-5) (22). We measured replication and rRNA degradation through the course of a one-step growth curve. The characteristic signature of RNase L activation in human cells is present during MERS-ΔNS4b and MERS-ΔNS3-5 infections at late time points (Fig. 8A) and can be quantified by Agilent RNA integrity number (RIN) and plotted over time (Fig. 8B). The characteristic rRNA degradation signature is not present during MERS-CoV infection or mock infection. We observed no reduction in titer for MERS-ΔNS4b but nearly a 10-fold reduction in titer for MERS-ΔNS3-5 compared with wild-type MERS-CoV infection (Fig. 8C). Thus, while MERS-CoV deletion mutants activate RNase L in Calu-3 cells, rRNA degradation was not very extensive compared to that observed in Sindbis virus infection of human A549 cells.

The more robust rRNA degradation seen with MERS-ΔNS3-5 than with MERS-ΔNS4b suggested that there may be additional mechanisms of RNase L antagonism by MERS-CoV. In fact, NS4a is a dsRNA binding protein that may help sequester viral dsRNA and prevent stimulation of OAS and subsequent activation of RNase L (17, 18). In addition, since NS4a and NS4b are expressed from the same mRNA by an unknown mechanism of initiation of translation, it is possible that deletion of ORF4b could result in increased expression of the upstream ORF encoding NS4a and increased dsRNA binding and inhibition of RNase L, negating the need for a PDE to antagonize RNase L. Thus, we constructed MERS-NS4b^H182R^, expressing NS4b with an inactive PDE, and assessed its replication and ability to active RNase L in Calu-3 cells. While MERS-NS4b^H182R^ replicated similarly to WT MERS (data not shown), it did promote RNase L degradation and like the deletion mutants only at late times postinfection (Fig. 8D).

Thus, MERS-CoV NS4b and BtCoV homologs have 2′,5′-PDE activity that antagonizes RNase L activation *in vitro* and is dependent on a catalytic His residue. In addition, like MHV NS2, lineage C *Betacoronavirus* NS4b activity is crucial for protection of rRNA integrity and for efficient replication of chimeric MHV in cell culture and in the case of MERS-CoV NS4b, in mice. While RNase L activation in Calu-3 cells is observed with both MERS-CoV deletion mutants, it is clearly less extensive than observed in the chimeric MHV infections of BMM and has less effect on viral replication as discussed below.

## DISCUSSION

Here we demonstrate that MERS-CoV and related BtCoV NS4b proteins inhibit the OAS-RNase L pathway by enzymatic cleavage of 2-5A, the activator of RNase L, potentially antagonizing IFN signaling. The type I IFN response is an essential component of the antiviral innate immune response and is activated when host sensors detect viral dsRNA, leading to production of type I IFN (9, 10, 24). IFN activates transcription of IFN-stimulated genes (ISG), leading to the development of an antiviral state in the infected cells and in neighboring cells, thereby restricting viral spread. Based on overexpression experiments, it was previously reported that MERS-CoV NS4b and HKU5 NS4b proteins antagonize type I IFN signaling by several different methods, including interaction with Tank binding kinase 1 (TBK1)/IκB kinase ε (IKKε), leading to inhibition of IFN regulatory factor 3 (IRF3) phosphorylation, nuclear localization, and subsequent beta interferon (IFN-β) transcription (16, 18, 19). It was recently shown by overexpression of NS4b and an IFN-β promoter reporter plasmid that MERS-CoV NS4b interacts with as yet unknown nuclear targets inhibiting IRF3- and IRF7-induced IFN-β transcription (19). It is important to note that there is little if any information on IFN antagonism during MERS-CoV infection, and none of these reports recognized the enzymatic activity of NS4b proteins. Nevertheless, the previous publications in combination with our current study of the enzymatic activity of NS4b proteins suggest that NS4b proteins are multifunctional with activities in both the cytoplasm and nucleus, consistent with its localization in both cellular compartments.

Searches for sequence homology between MERS-CoV NS4b in protein databases were unsuccessful. However, NS4b was predicted by tertiary structural modeling to be a 2H-PE and the fact that it is conserved in all sequenced lineage C *Betacoronavirus* genomes suggested that NS4b is an enzymatically active 2′,5′-PDE (29), as confirmed in this study. Furthermore, NS4b protein antagonizes the OAS-RNase L pathway by cleaving 2-5A and blocking the subsequent activation of RNase L (7, 29). Additionally, closely related lineage C *Betacoronavirus* BtCoV-SC2013 and BtCoV-HKU5 NS4b proteins are enzymatically active PDEs with similar activity to MERS-CoV NS4b. We have demonstrated that these lineage C proteins can cleave 2-5A *in vitro* and that their kinetic parameters are similar to those of NS2.

Limitation of RNase L activation can have a profound effect on enhancing virus replication and spread as well as leading to decreased IFN production in myeloid cells (7, 29, 42). Indeed, we previously found that the 2′,5′-PDE of MHV NS2 is a determinant of myeloid cell tropism and a critical liver virulence factor in mice (29). Thus, to initially assess the effectiveness of NS4b proteins, we utilized the chimeric MHV NS2 mutant virus system that was previously used to demonstrate activity for RVA and AKAP PDEs (28, 34). Lineage C PDEs were expressed from chimeric MHV with an NS2^Mut^ background, encoding an inactive PDE. MERS-CoV NS4b and BtCoV homologs were detected by immunoblotting to different extents, as normalized to the amount of MHV nucleocapsid protein detected, with the mutant proteins less well expressed compared to WT proteins. The different levels of expression of WT proteins and the more efficient expression of the WT PDEs compared to the corresponding mutant proteins was observed in both BMM and murine 17Cl-1 fibroblasts (Fig. 5 and data not shown), suggesting that there may be differences in stability in the WT proteins and that the mutant proteins may be less stable. Nevertheless, we were able to rescue replication in murine BMM with wild-type MHV-MERS, wild-type BtCoV-2013 (Fig. 6), and wild-type BtCoV-HKU5 (data not shown) but not with viruses expressing corresponding mutant PDEs and in the case of MHV-MERS^WT^, but not MHV-MERS^Mut^, *in vivo* in the mouse liver as well. Since the NS4b catalytic mutants used here are poorly expressed, they do not provide the ideal control and we cannot rule out the possibility that either one or more would rescue at a higher expression level or that factors other than the PDE activity contribute to rescue. This, however, is highly unlikely, as we know from several previous studies (28, 29, 34) that efficient expression of catalytic mutant PDE does not rescue replication of MHV NS2 mutant virus, while supplying with heterologous PDE does (28, 29, 34).

We have previously demonstrated that cytoplasmic localization of PDE activity is necessary to antagonize the OAS-RNase L pathway. Murine AKAP7, which contains an NLS, can rescue only chimeric PDE-deficient MHV in the absence of the NLS (34). Interestingly, it is unnecessary to remove the NLS from the MERS and SC2013 NS4b proteins to achieve rescue of chimeric MHV, and in fact, removal of the N-terminal 52 amino acids, including the NLS, of MERS-CoV NS4b confers no replication enhancement (Fig. 6), suggesting that the NLS neither hinders nor improves OAS-RNase L antagonism. Recombinant HKU5 NS4b clearly has 2′,5′-PDE activity (Fig. 3), and MHV-HKU5^WT^ (but not MHV-HKU5^Mut^) clearly replicates efficiently in B6 BMM (data not shown), demonstrating that HKU5 NS4b can replace the function of NS2 in the MHV chimeric background. However, there was still detectable RNA cleavage in BMM infected with both MHV-HKU5^WT^ and MHV-HKU5^Mut^ (data not shown). The most likely explanation is that BtCoV-HKU5 NS4b does not antagonize RNase L activation as well as MERS-CoV or BtCoV SC2013 NS4b does, because it is more localized to the nucleus, consistent with our findings with the PDE of AKAP7 (34). It is also possible the rRNA is more susceptible to cleavage than viral RNA; therefore, the less effective BtCoV-HKU5 NS4b cannot antagonize RNase L effectively enough to spare rRNA while still protecting viral and/or cellular mRNA.

We have demonstrated that MERS-CoV antagonizes OAS-RNase L activation in Calu-3 cells and that mutant viruses with deletions of either NS3 to NS5 or NS4b alone as well as the catalytic mutant MERS-NS4b^H182R^ activate this pathway. Although all mutants induced rRNA degradation, only MERS-ΔNS3-5 had a reduction in viral titer by 24 h postinfection. Thus, the lack of effect on replication of MERS-ΔNS4 or MERS-NS4b^H182R^ correlates with the weak rRNA degradation observed and indicates a lack of biological effect in this cell type of deleting or inactivating the NS4b PDE. This may be due in part to the very late times postinfection (24 to 48 h) that we observe rRNA degradation; virus replication is already quite robust by that time, and it may be too late for restriction of replication to occur.

We have found previously that cell type is critical for robust activation of RNase L and reduction in viral titers during infection of murine cells and have observed activation of RNase L by MHV^Mut^ only in myeloid cells (43) and endothelial cells (unpublished data). The use of Calu-3 epithelial airway cells may explain the lack of restriction of MERS-ΔNS4b replication and only 10-fold reduction for MERS-ΔNS3-5, when in murine BMM, abrogation of RNase L antagonism by MHV, which is observed by 9 h postinfection, leads to 100- to 1,000-fold reduction in viral titers. Indeed, RNase L is not readily activated in Calu-3 cells compared to other cell types; the amount of rRNA degradation observed in Sindbis virus-infected A549 cells is clearly more than in Calu-3 cells (Fig. 8A), and we did not observe robust activation of RNase L Calu-3 cells even when transfected with the dsRNA surrogate poly(I ⋅ C) (data not shown). Further studies are being directed at identifying a cell type in which PDE activity enhances viral replication.

## MATERIALS AND METHODS

### Cell lines and mice.

Murine L2 (L2) and baby hamster kidney cells expressing MHV receptor (BHK-MHVR) were cultured as described previously (7, 44). Calu-3 clone 2B4 cells (a kind gift from Chien-Te Tseng, University of Texas Medical Branch, Galveston, TX) were cultured with Gibco minimum essential medium (MEM) (Thermo Fisher Scientific, Grand Island, NY) supplemented with 20% fetal bovine serum (FBS) (HyClone, GE Healthcare Bio-Sciences, Pittsburg, PA), penicillin-streptomycin (Gibco), and amphotericin B (Fungizone) (Gibco). Vero CCL-81 cells (ATCC, Manassas, VA) were cultured, and the MERS-CoV plaque assay was performed as previously described (22). Human A549 cells were cultured in RPMI 1640 medium (Gibco) supplemented with 10% FBS, 100 U/ml of penicillin, and 100 µg/ml streptomycin. All cells were maintained at 37°C and 5% CO_2_. B6 mice and RNase L^−/−^ mice were bred and maintained in the University of Pennsylvania animal facility, and the protocols were approved by the Institutional Animal Care and Use Committee at the University of Pennsylvania. Primary BMM were derived from bone marrow harvested from the hind limbs (tibia and femur) of 4- to 6-week-old B6 or RNase L^−/−^ mice and described previously (29, 45). Cells were cultured in Dulbecco modified Eagle medium (DMEM) (Gibco) supplemented with 10% FBS (HyClone) and 20% L929 cell-conditioned media for 6 days before infection.

### Plasmids and recombinant viruses.

NS4b sequences for MERS-CoV (GenBank accession no. AFS88939.1), SC2013 (GenBank accession no. AHY61340.1), and HKU5 (accession no. YP_001039965.1) were obtained from NCBI. NS4b cDNA sequences were synthesized with the addition of a 5′ SalI site and 3′ Flag epitope and NotI site and then cloned into pUC57 (Bio Basic, Markham, Ontario, Canada). Site-directed mutagenesis was performed on each NS4b plasmid to generate point mutants (CAC to CGC) containing histidine-to-arginine mutations within the second catalytic motif yielding H182R, H186R, and H183R for MERS-CoV, SC2013, and HKU5 NS4b proteins, respectively, and subsequently sequence verified. Each NS4b gene was then PCR amplified and subcloned in frame with maltose binding protein into pMAL protein expression vector. MHV NS2 and NS2^H126R^ were previously cloned into pMAL parallel-2 vector (29).

MHV^WT^, MHV^Mut^, and all chimeric viruses were constructed with the MHV strain A59 infectious clone (44). Each WT or mutant NS4b cDNA was subcloned into open reading frame 4 (ORF4) of the infectious clone plasmid G_EGFP_ (EGFP stands for enhanced green fluorescent protein), and sequences were confirmed as described previously (28). The full-length cDNAs were assembled with A to E (MHV^WT^), F (MHV^Mut^), and G (NS4b) fragments, and RNA was *in vitro* transcribed and recovered as previously described (44). Briefly, infectious clone fragments were excised from their respective plasmids by restriction enzyme digestion. The fragments were gel purified and ligated *in vitro* to create a full-length cDNA. cDNA was *in vitro* transcribed with mMessage mMachine T7 transcription kit (Ambion; Thermo Fisher, Grand Island, NY), generating full-length genomic RNA. Each genomic RNA transcript was split into two distinct pools and electroporated into BHK-MHVR along with N-protein transcript using Gene Pulser II (Bio-Rad, Hercules, CA). Electroporated cells were incubated until cytopathology was evident throughout, freeze thawed three times, and plaque purified, and one plaque from each pool was utilized in parallel for all assays.

MERS-ΔNS4b was constructed using the MERS-CoV infectious clone (22). To delete NS4b, sequence was synthesized (Bio Basic) in which mutations were made in the overlap region between NS4a and NS4b to abrogate putative start codons (T2C and T17C; all positions relative to the NS4b start) and to insert premature stop codons (A91T, C97A, and C102G). Additionally, residues 106 to 669 were deleted to remove the majority of NS4b but leave in place transcriptional regulatory sequence 5. The truncated NS4b cassette and MERS-CoV infectious clone F plasmid were digested with PacI and SanDI, and truncated NS4b was ligated into the MERS-CoV F plasmid. To construct the MERS-NS4b^H182R^ mutant, two PCRs were performed using the F plasmid as the template to synthesize overlapping DNA fragments and introducing an H182R substitution. These two templates were then joined in an overlapping extension PCR. The resultant product was digested with PacI and SanDI and cloned into the MERS F plasmid. Assembly of the infectious clone and recovery of infectious virus were performed as described previously (22). The potential gain-of-function (GOF) concerns of MERS-ΔNS4b and MERS-NS4b^H182R^ were evaluated and reviewed by NIH under grant U19AI107810 and approved for continued study. The MERS-ΔNS3-5 mutant (22) was generated prior to GOF regulations. The MERS-ΔNS3-5 and catalytic mutant display attenuated growth on select interferon-competent human cell lines.

### FRET assay and enzyme kinetics.

NS4b of MERS-CoV, SC2013, and HKU5 were cloned into pMal parallel-2 vector (46) and expressed in BL21 T7 express competent *E. coli* (NEB, Inc., Ipswich, MA) as maltose binding protein (MBP) fusion proteins and purified by affinity chromatography followed by ion exchange chromatography on MonoQ GL10/100 using a NaCl gradient from 0 to 1 M in 20 mM HEPES (pH 7.2) and gel filtration in 20 mM HEPES (pH 7.2) containing 100 mM NaCl as described earlier for purification of MHV NS2 (29). Briefly, purified proteins (10 µM MBP as control or 1 µM concentration of MBP fusion proteins with NS4b of MERS-CoV, SC2013, and HKU5 or their catalytically inactive mutants described in the previous section) in 150 µl of assay buffer (20 mM HEPES [pH 7.2], 10 mM MgCl_2_, 1 mM dithiothreitol) were incubated at 30°C with various concentrations of (2′-5′)p_3_A_3_. Aliquots of 20 µl were withdrawn at different times, and the reactions were stopped by heat inactivation at 95°C for 3 min followed by 30-min centrifugation at 20,000 × *g* (4°C) and supernatants were carefully removed. A fluorescence resonance energy transfer (FRET)-based RNase L activation assay was used to determine enzyme kinetics by measuring the uncleaved, intact (2′,5′)p_3_A_3_ as previously described (47). The amounts of 2-5A cleaved were determined by the relative fluorescence units (RFU) using standard curves with different concentrations of authentic 2-5A. The reaction velocities were determined by nonlinear fit of the data. The *k*_cat_ and *K_m_* were determined in GraphPad Prism 5.0 for Windows by plotting velocity against substrate concentrations, and data were analyzed by the following equation: *Y* = Et × *k*_cat_ × [*X*/(*K_m_* + *X*)], where *Y* is the velocity of the reaction in micromolar per second, *X* is the substrate concentration in micromolar, and Et is the concentration of enzyme catalytic sites. The kinetics were determined in triplicate reactions.

### *In vitro* infections. (i) Chimeric MHV infections.

BMM were mock inoculated or inoculated with MHV^WT^, MHV^Mut^, MHV-MERS^WT^, MHV-MERS^Mut^, MHV-SC2013^WT^, MHV-SC2013^Mut^, MHV-HKU5^WT^, or MHV-HKU5^Mut^. Virus was added to cells at a multiplicity of infection (MOI) of 1 PFU/cell and allowed to adsorb for 1 h at 37°C. Cultures were washed with phosphate-buffered saline (PBS) (three times) and fed with medium. At the times indicated in the figures, cells were fixed and analyzed for protein expression by immunofluorescent staining, lysed, and analyzed for protein expression by immunoblotting or analyzed for degradation of RNA, or supernatants were harvested for quantification of viral titers by plaque assay on L2 cells (48).

### (ii) MERS-CoV infections of Calu-3 cells.

Prior to infection, Calu-3 cells were washed once with PBS (Gibco) to remove residual fetal bovine serum (FBS). Cells were inoculated under biosafety level 3 (BSL3) conditions with MERS-CoV, MERS-ΔNS4b, MERS-NS4b^H182R^, or MERS-ΔNS3-5 at an MOI of 1 PFU/cell and allowed to absorb for 40 min at 37°C. The inoculum was then removed, and the cells were washed twice with PBS prior to adding media containing low FBS (4%). At the time points indicated in the figures, 100 µl of medium was collected and subsequently analyzed by plaque assay in Vero CCL-81 cells as previously described (22), and total RNA was harvested in 1 ml Trizol.

### Immunoblotting.

At 12 h postinfection, cells were lysed in Nonidet P-40 (NP-40) buffer (1% NP-40, 2 mM EDTA, 10% glycerol, 150 mM NaCl, and 50 mM Tris [pH 8.0]) containing protease inhibitors (Roche). Protein concentrations were measured using a DC protein assay kit (Bio-Rad). Supernatants were mixed 3:1 with 4× SDS-PAGE sample buffer. Samples were boiled, separated by 4 to 15% SDS-PAGE, and transferred to polyvinylidene difluoride (PVDF) membranes. Blots were blocked with 5% nonfat milk and probed with the following antibodies: anti-Flag M2 mouse monoclonal antibody (Agilent) (1:1,000), anti-N mouse monoclonal antibody (a gift from Julian Leibowitz) (1:400), and anti-GAPDH mouse monoclonal antibody (Thermo Fisher Scientific) (1:1,000). Anti-mouse secondary antibodies labeled with horseradish peroxidase (HRP) (Santa Cruz; 1:5,000) were used to detect the primary antibodies. The blots were visualized using SuperSignal West Dura extended-duration substrate (Thermo Scientific). Blots were probed sequentially with antibodies with the blots stripped between antibody treatments.

### Indirect immunofluorescence assay.

L2 cells were infected at an MOI of 1 PFU/cell for 8 h with MHV^WT^, MHV-MERS^WT^, MHV-SC2013^WT^, or MHV-HKU5^WT^. Cells were washed with Dulbecco’s phosphate-buffered saline (DPBS) (Gibco) and then fixed with 4% paraformaldehyde in DPBS (Gibco). After fixation, cells were permeabilized with 0.1% Triton in DPBS (Gibco) and then blocked with bovine serum albumin. Cells were then stained with mouse monoclonal anti-nucleocapsid (1:200) and rabbit anti-Flag polyclonal (Sigma) (1:2,000) antibodies. After the cells were washed, secondary staining with Alexa Fluor 488-labeled goat anti-mouse (1:400) and Alexa Fluor 594-labeled goat anti-rabbit (1:1,000) antibodies was performed. The cells were washed once more, and the nuclei were then stained with 4′,6′-diamidino-2-phenylindole (DAPI). Stained cells were imaged at a magnification of ×400 on an Eclipse TE2000-U fluorescence microscope (Nikon Instruments, Inc.). Images were acquired using NIS-Elements Basic Research microscope imaging software (Nikon Instruments, Inc.). This was performed at least twice for all viruses.

### Qualitative and quantitative analyses of RNase L-mediated rRNA degradation.

RNA from B6 BMM infected with MHV^WT^, MHV^Mut^, MHV-MERS^WT^, MHV-MERS^Mut^, MHV-SC2013^WT^, MHV-SC2013^Mut^, MHV-HKU5^WT^, or MHV-HKU5^Mut^ was harvested with an RNeasy kit (Qiagen, Valencia, CA) 15 h postinoculation. Alternatively, Calu-3 cells infected with MERS-CoV, MERS-ΔNS4b, MERS-ΔNS3-5, or MERS-NS4b^H182R^, and at the time points indicated in the figures, RNA was harvested with Trizol (Sigma Aldrich, St. Louis, MO), extracted with chloroform (Sigma), and precipitated with isopropanol (Sigma). RNA was then separated and analyzed on RNA nanochips with an Agilent Bioanalyzer 2100 (Agilent Technologies, Santa Clara, CA), and the pseudogels were normalized for concentration (49). RNA integrity numbers (referred to as RIN values) (50) are measurements of RNA integrity produced by the Agilent Bioanalyzer. RNA from A549 cells infected with Sindbis virus (51) was used as a marker for the pattern of human RNase L-dependent rRNA cleavage products.

### Inoculation of mice.

Four-week-old B6 or RNase L^−/−^ mice were anesthetized with isoflurane (Abbott Laboratories, Chicago, IL) and inoculated intrahepatically with MHV^WT^, MHV^Mut^, MHV-MERS^WT^, or MHV-MERS^Mut^ in 50 µl of DPBS (Gibco) containing 0.75% bovine serum albumin (Sigma). Mice were humanely euthanized with CO_2_ and perfused with DPBS (Gibco), and their livers were harvested at day 5 postinoculation. Viral titers were determined by plaque assay of lysates (48). This study was carried out in strict accordance with the *Guide for the Care and Use of Laboratory Animals* (41) and the Public Health Service Policy on Humane Care and Use of Laboratory Animals (52).
